# Operational Research to Support Rapid Evidence-Based Responses to Outbreaks: Learnings from COVID-19

**DOI:** 10.4269/ajtmh.23-0893

**Published:** 2024-10-08

**Authors:** Anne Hoppe, Pallavi Dani, Grace Mwangoka, Stephen Vreden, Guillaume Breton, Jerome Ateudjieu, Joaniter I. Nankabirwa, Júlia Sambo, Rose Masaba, Tatenda Maparo, Goodman Sibeko, Richard Njouom, Boris Tchounga, Isaac Ssewanyana, Chancy Chavula, Lindiwe Nchimunya, Tatiana Djikeussi, Sam Accellam, Hedley Cairo, David Walcott, Aamir J. Khan, Shaukat Khan, Daniel G. Bausch

**Affiliations:** ^1^ FIND, Geneva, Switzerland;; ^2^ Technical Strategy and Innovation, Elizabeth Glaser Pediatric AIDS Foundation, Geneva, Switzerland;; ^3^ Biomedical Research and Clinical Trials, Ifakara Health Institute, Bagamoyo, Tanzania;; ^4^ Foundation for the Advancement of Scientific Research, Paramaribo, Suriname;; ^5^ Technical Department, Solthis, Paris, France;; ^6^ Department of Health Research and Intervention, M. A. SANTE, Yaoundé, Cameroon;; ^7^ Research Department, Infectious Diseases Research Collaboration, Kampala, Uganda;; ^8^ Health Research Management & Coordination, Instituto Nacional de Saude, Maputo, Mozambique;; ^9^ Research, Elizabeth Glaser Pediatric AIDS Foundation, Nairobi, Kenya;; ^10^ Access Program, Clinton Health Access Initiative, Harare, Zimbabwe;; ^11^ Psychiatry and Mental Health, University of Cape Town, Cape Town, South Africa;; ^12^ Department of Virology, Centre Pasteur of Cameroon, Yaoundé, Cameroon;; ^13^ Research, Elizabeth Glaser Pediatric AIDS Foundation, Yaoundé, Cameroon;; ^14^ Laboratory Services, Central Public Health Laboratories, Kampala, Uganda;; ^15^ Diagnostics, Clinton Health Access Initiative, Lilongwe, Malawi;; ^16^ Diagnostics, Clinton Health Access Initiative, Lusaka, Zambia;; ^17^ Malaria Program, Ministry of Health Suriname, Paramaribo, Suriname;; ^18^ Department of Microbiology, University Hospital Of The West Indies, Kingston, Jamaica;; ^19^ Infectious Diseases, IRD Global, Singapore, Singapore;; ^20^ Global Diagnostic Teams, Clinton Health Access Initiative, Boston, Massachusetts

## Abstract

During the COVID-19 pandemic, the need for making testing readily available was recognized as an important factor for individuals to help make informed decisions, including to isolate or seek care, and for policymakers to control transmission. Toward this end, FIND and the Access to COVID-19 Tools Accelerator funded 16 rapid operational research studies and one implementation project in Africa, the Caribbean, and Asia evaluating the utility, acceptability, and feasibility of different community-based SARS-CoV-2 testing approaches. Here, we discuss common factors and challenges encountered during study implementation. We note six key factors essential for success: 1) collaboration and partnerships; 2) buy-in of local stakeholders, including communities; 3) access to affordable supplies; 4) flexible financing; 5) effective approval systems; and 6) a skilled and motivated workforce. We also note various challenges that must be addressed to fully capitalize on these success factors. In particular, ethics committees are often not well equipped to assess operational research during outbreaks. Outbreaks, especially of novel pathogens, are unpredictable, and transmission dynamics are even more likely to change if the pathogen is prone to frequent mutations, such as SARS-CoV-2. Research that aims to evaluate strategies for curbing transmission must hence be easily and swiftly adaptable. This requires flexibility from researchers, funders, staff conducting the studies, and ethics and other approval committees. International guidelines for evaluating operational research protocols in outbreaks are needed to provide timely evidence to enable informed decisions by individuals, communities, and policymakers, thereby reducing both the human and the economic impact of outbreaks.

## INTRODUCTION

Pathogens have accompanied human populations and caused various epidemics throughout history, yet the frequency of these outbreaks has increased and is expected to rise further as a result of globalization, increased population density, more human-animal interactions, and climate change.^1^^,^^2^ It is therefore critical that we capitalize on the lessons learned during the coronavirus disease 2019 (COVID-19) pandemic and apply its general learnings to localized outbreaks, thereby reducing the spread of disease.

**Figure 1.**
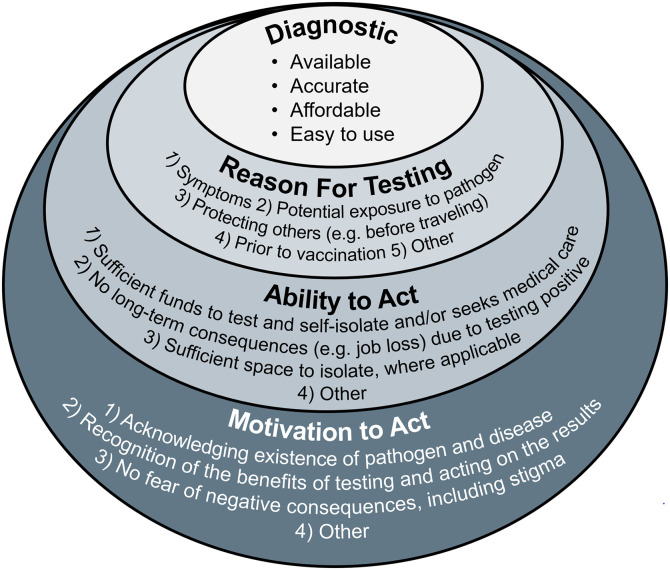
Four key criteria for mitigating the spread of an infectious disease with known pathogens and transmission patterns.

As with any pathogen with outbreak potential, early identification of the pathogen, knowledge about the pathogenesis and its transmission dynamics, and swift implementation of appropriate control measures are key for preventing the spread of disease. For COVID-19, at least four criteria had to be met for this approach to be effective: 1) availability of an accurate, easy to use, affordable diagnostic test; 2) defining indications for testing for the pathogen; 3) ability to test, self-isolate, or seek medical care, as applicable; and 4) willingness to test, self-isolate, or seek medical care, as applicable (Figure 1).

**Figure 2.**
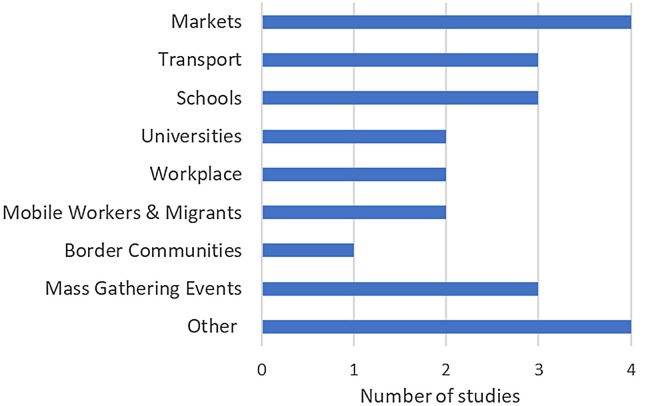
Communities in which SARS-CoV-2 testing was performed. SARS-CoV-2 = severe acute respiratory syndrome coronavirus 2.

During the early stages of the COVID-19 pandemic, there was a strong focus on diagnosis and testing within healthcare facilities and less on a community-centered approach. As the pandemic progressed and with the availability of antigen rapid diagnostic tests (Ag-RDTs), the need for making testing more readily available was recognized as an important factor for individuals to evaluate their status and make informed decisions, including isolating and seeking care. To this effect, the nonprofit organization FIND (an international organization promoting diagnostic development and access in low-and middle-income countries [LMICs], based in Geneva, Switzerland)^3^ and the Access to COVID-19 Tools Accelerator (ACT-A) partnership^4^ commissioned 16 rapid operational research (OR) studies and one implementation project on community-based severe acute respiratory syndrome coronavirus 2 (SARS-CoV-2) testing approaches in LMICs through a competitive request for proposals. The studies took place on three continents and 13 countries and focused on systematic testing within a diverse set of communities (Figure 2; Supplemental Table 1). All aimed to provide actionable evidence to local health authorities and policymakers on the most effective use of Ag-RDTs to ensure rapid detection of and swift response to SARS-CoV-2 in community settings. Detailed results of these studies are presented in this journal supplement.^5^^–^^18^

**Figure 3.**
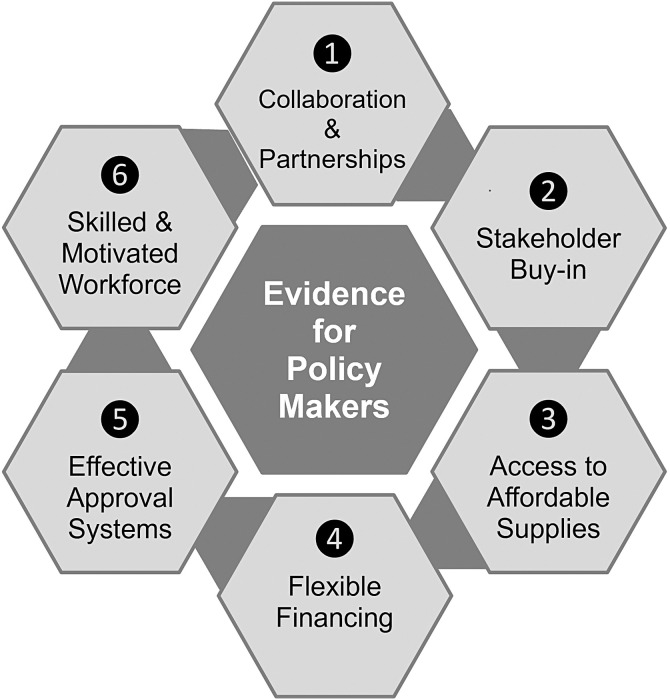
Key success factors for OR. OR = operational research.

Key success factors and challenges were recorded throughout the different phases of each study. These were discussed by senior representatives of study teams and FIND’s COVID-19 OR team during a two-day meeting in Kigali, Rwanda, in December 2022. Here, we report six key success factors (Figure 3) and challenges that were common across all of the studies. The challenges should be addressed for future OR to provide timely and conclusive evidence to policymakers, and we make several recommendations toward that goal.

## SIX CRITICAL SUCCESS FACTORS

### 1. Collaboration and partnerships.

More than 70% of the studies were developed in partnership between different organizations, ensuring a complementary approach to skill sets and competencies. The most frequent grantees were international nongovernmental organizations (INGOs) (*n =* 8), local organizations (*n =* 6), and Ministries of Health (MoHs) (*n =* 4). Ministries of Health and MoH-associated organizations were involved in 12/17 (71%) studies, including 7/8 (89%) of the proposals led by INGOs. This ensured that studies were aligned with MoH priorities for tackling the COVID-19 pandemic and allowed for additional health-related messaging to be incorporated as needed (Box 1).^9^^,^^15^ Academic institutions were involved in ten different studies,^5^^–^^7^^,^^10^^–^^13^^,^^16^^–^^18^ and two of the study teams collaborated with community-based organizations.^9^^,^^16^

Box 1 **Alignment with MoH priorities presents opportunities**

- **The MoH conducting research to prevent the spread of COVID-19 in Mozambique**
  The National Institute of Health in Mozambique assessed the effectiveness of a walk-through COVID-19 testing approach for reaching high-risk populations that do not attend health facilities. Overall, 4,453 individuals were tested at walk-through testing stations in markets and ports. Seventy percent of individuals were fully vaccinated.^15^
- **Incorporating messaging relating to an Ebola virus outbreak into SARS-CoV-2 awareness campaigns**
  The Central Public Health Laboratory and the governing body of Kampala, Uganda, evaluated the feasibility, utility, and acceptability of SARS-CoV-2 Ag-RDTs in markets and trade hubs. Overall, 13,086 volunteers were tested during four rounds of SARS-CoV-2 screening. The final round of testing in September 2022 coincided with the beginning of an Ebola virus outbreak in Uganda, and messaging related to that outbreak was hence incorporated in the study’s awareness campaigns.^9^

The funder, FIND, in collaboration with the ACT-A, actively engaged with the different study teams during the design stage to ensure that study designs were stringent and able to meet the objectives. FIND also provided continuous guidance during the implementation phases, as needed, often facilitating the work in the context of continuously evolving waves of COVID-19 transmission and keeping abreast of learnings during the course of the studies to allow the necessary flexibility. However, neither FIND nor ACT-A influenced the analyses or conclusions drawn from any of the work conducted.

### 2. Stakeholder buy-in.

For OR to be effectively implemented, it must not only engage with key policymakers but also incorporate extensive community engagement. It is essential that the testing approach and any interventions be locally driven^19^^,^^20^ and that both long- and short-term consequences are carefully assessed. For instance, although strictly enforced home- or facility-based isolation of any COVID-19–positive persons may prevent virus transmission in the short-term, the negative consequences of enforced isolation can be detrimental for those who are dependent on daily wages.^21^ In the long-term, the fear of enforced isolation can cause resistance to getting tested, thereby leading to delayed detection of a localized outbreak. Furthermore, to avoid testing fatigue, the frequency of testing must be carefully balanced against the estimated risk of infection.^6^

As noted above, although MoHs were co-applicants on more than 70% of the research proposals, community stakeholders were unfortunately not equally engaged across all studies. When community engagement activities did take place, these were seen as a critical success factor for creating awareness of studies, debunking myths related to studies and COVID-19, building trust, and ensuring the safety and security of staff and participants.^5^^,^^6^^,^^9^^,^^12^^–^^14^^,^^16^^–^^18^ In general, engagement activities included meetings with key community members and leaders (Box 2),^5^^,^^7^^,^^8^^,^^10^^,^^18^ involving community champions, distribution of flyers, and use of speaker phones on days when testing was conducted.^8^^–^^10^^,^^12^^,^^15^^–^^17^ For those studies taking place in school or university settings^5^^–^^7^ and evaluating self-testing,^18^ more extensive stakeholder engagement activities were conducted (Box 2C–E).

Box 2 **Key stakeholders engaged as part of different OR studies**

- The M. A. SANTE team in Cameroon enrolled 9,594 passengers in six intercity bus travel agencies to assess three different strategies for preventing the spread of SARS-CoV-2 during travel. The Ministries of Public Health and Transport and intercity travel agencies supported the study, and the study’s findings were presented to all key stakeholders.^10^
- The Clinton Health Access Initiative (CHAI) tested 4,071 adults in marketplaces in Malawi and Zambia for SARS-CoV-2, demonstrating that community testing is feasible and acceptable. The implementation of the study was facilitated through extensive stakeholder engagement, including units of the MoH, district health offices, city councils, chiefs, communities, and market committees. Through these engagements, the team secured 2,000 COVID-19 Ag test kits from the MoH, space for conducting the testing, and on-site support from members of the market health committee^8^.
- The Infectious Diseases Research Collaboration, Uganda, conducted monthly cross-sectional surveys in 11 schools in two border districts. The study team engaged with Ministry of Education, heads of schools, teachers, parent/teachers’ associations, and parents before and throughout the duration of this study. Overall, 8,902 students were recruited over the course of 4 months.^5^
- The team of the Center Pasteur of Cameroon conducted an OR study in six Cameroonian state universities to detect SARS-CoV-2 cases in these settings and to assess COVID-19–related knowledge, attitudes, and practices. The team engaged with deans, rectors, senior staff, and student delegates as well as the MoH through meetings prior to project commencement. The study recruited 7,006 participants. Free alcohol gel and face masks were considered a motivating factor.^7^
- The team at the Ifakara Health Institute evaluated the acceptability, feasibility, and uptake of Ag-RDT self-testing at the community level in Tanzania to allow expansion of testing services to hard-to-reach communities. Prior to the start of the study, the research team engaged with the MoH’s National COVID-19 task force, local government, health management teams, and community leaders. Their recommendations were incorporated and included moving testing sites to different locations to align with anticipated demands. Overall, 448 individuals agreed to be tested, with 148 (33%) opting for self-testing.^18^

Involvement of community organizations at the study design stage helped to address concerns of community members and hence increase acceptability of testing. Information regarding concerns of community members that might pose obstacles to testing was obtained as part of various studies (Box 3)^16^ and included COVID-19 fatigue, feeling healthy, time required for testing, already tested/vaccinated, myths about COVID-19, the belief that testing by nasal swabs would be painful, skepticism about the reliability of the test, and fear of negative consequences.

Box 3 **Concerns affecting uptake of SARS-CoV-2 screening in South Africa**

In South Africa, more than 25% of people who were invited for community-based COVID-19 screening declined participation in IRD’s OR study. Seven hundred of these individuals were surveyed, and lack of time (38%), not interested (27%), already tested, and vaccinated (25%) were given as main reasons for refusing participation in this study. Of those who agreed to participate in the study, not being comfortable with procedures (52%) and lack of time (42%) were given as predominant reasons for declining COVID-19 testing. Identifying and addressing these concerns helped to increase participation in the study and the uptake of testing services.^16^

As expected, participation was higher in settings where incentives for testing, such as distribution of hand gels,^10^ school materials, and visit reimbursement,^5^ were provided. Also, although linking testing to vaccination increased uptake in Kenya,^13^ the two activities needed to be clearly delinked in settings with high skepticism toward the vaccine, such as Uganda and the low-income communities in Jamaica.^6^^,^^9^ In Mali and Suriname, the integration of malaria and SARS-CoV-2 testing, respectively, was a key factor for its acceptability and uptake (Box 4).^14^^,^^17^^,^^22^

Box 4 **Successful integration of community-based malaria and SARS-CoV-2 testing**

- In Mali, awareness and perceived risk of contracting COVID-19 were low in rural areas. In a study conducted by Solthis, community health workers systematically tested patients with COVID-19 symptoms for SARS-CoV-2 and, when fever was present, malaria using Ag-RDTs. Integrating COVID-19 testing with malaria testing aligned with the needs of patients and increased acceptability. The community-based testing strategy with testing for both SARS-CoV-2 and malaria using Ag-RDTs was more effective than the national malaria control strategy.^17^
- Suriname’s remote gold mining communities have long been sources of malaria transmission. In its fight against malaria, the MoH’s Malaria Program successfully trained members of these communities to locally provide malaria test and treatment services. During the COVID-19 pandemic, these malaria workers proved instrumental in also providing COVID-19 test services.^14^^,^^22^

### 3. Access to affordable supplies.

During the COVID-19 pandemic, the health community supported the fastest, most coordinated global effort in history to apply testing to a major pandemic.^4^ As a result, diagnostics such as nucleic acid amplification tests and Ag-RDTs were rapidly developed and deployed. In part through the efforts of the diagnostics pillar of the ACT-A, close to 170 million tests were procured for LMICs, nearly $1 billion USD was awarded to LMICs for the procurement of diagnostics, and manufacturer’s costs of SARS-CoV-2 Ag-RDTs were halved to less than $2.50 USD.^23^

Despite these efforts, access to diagnostics and other supplies was not equitable across and within countries. The supply of Ag-RDTs of at least 3/16 studies was negatively impacted by large orders placed by the United States. It is hoped that strong political will and postpandemic investments to increase local manufacturing capabilities for vaccines, diagnostics, treatments, and personal protective equipment will eventually reduce the differences in purchasing powers.^24^^–^^28^ However, it remains to be seen whether these will reduce the costs per Ag-RDT, as many manufacturers have raised concerns with regard to just-in-time manufacturing and economies of scale.

In the studies published in this supplement, the costs per Ag-RDT varied from $2.60 to $5 USD (median, $3.25 USD; mean, $3.50 USD) owing to additional supply chain–related costs. These costs increased significantly when costs for personnel, other supplies, and essential activities were added (Box 5).^13^ The large-scale deployment of these tests placed a significant burden on health systems and, where testing could not be offered for free, on individuals. In Africa, almost 80% of adults earn their living in the informal sector and do not receive a regular fixed wage^29^; many of these people are dependent on a daily income and, as such, are unlikely to cease their daily activities if they experience mild symptoms or are asymptomatic. Furthermore, it has been estimated that roughly one-third of Africa’s population (around 489 million people) were living below the extreme poverty line of $1.90 USD a day in 2021, and this number is not likely to have decreased significantly in 2022.^30^ These individuals can likely only afford and will only seek testing once it is absolutely essential, likely in the latter stages of disease when they may have already transmitted the virus to many others.

Box 5 **Cost efficiency of SARS-CoV-2 community testing**

Mass testing with Ag-RDTs, including testing of asymptomatic individuals, requires a large investment in both personnel and financial resources. The Elizabeth Glaser Pediatric AIDS Foundation estimated the costs from a health systems perspective using a micro-costing method, combining top-down and bottom-up approaches to obtain resource use and costs per line item. In Kenya, the major cost driver was community mobilization followed by purchase of SARS-CoV-2 Ag-RDT, personnel, meetings, travel and transport, supplies, and capital costs of equipment. Although the cost of a test kit was $3.25 USD, the cost per individual tested was $15.89 USD, and the cost per new COVID-19 case detected was $1,484 USD. Analyses of program inputs are a useful tool to identify main cost drivers, inform planning, and improve resource allocation for mass SARS-CoV-2 Ag-RDTs in community settings.^13^

### 4. Flexible financing.

Overall, there was great appreciation by project partners for funding to be available for these short studies with unavoidably unpredictable impact, for the rapid process of allocating funding, and for the swift decision and approval process by FIND for any reallocations. However, various challenges were encountered with regard to financing: The funding cycle for the studies was from January to December 2022. All studies were designed between quarter 4 of 2021 and early quarter 1 of 2022, during the peak of the COVID-19 omicron wave, and were implemented in quarter 2 and quarter 3 of 2022, when SARS-CoV-2 transmission was waning in many parts of the world.^31^ The timing of the implementation phases of the different studies thus coincided both with low transmission rates and significant COVID-19 fatigue. Budget reallocations were often required and were made owing to changes in the transmission dynamics as well as local guidelines, but were nevertheless limited by the short funding and implementation cycle designed to rapidly collect actionable evidence in the face of a pandemic. The unavoidable failure to meet target case numbers, and thus statistical endpoints, in many of the studies often impeded making conclusive evaluations of the impact of the interventions on transmission and furthermore increased the cost of each COVID-19 case detected and averted through the intervention. A flexible funding cycle that would have allowed for the implementation phase to coincide with the start of a new wave of transmission would have been beneficial.

### 5. Effective review and approval systems.

**Figure 4.**
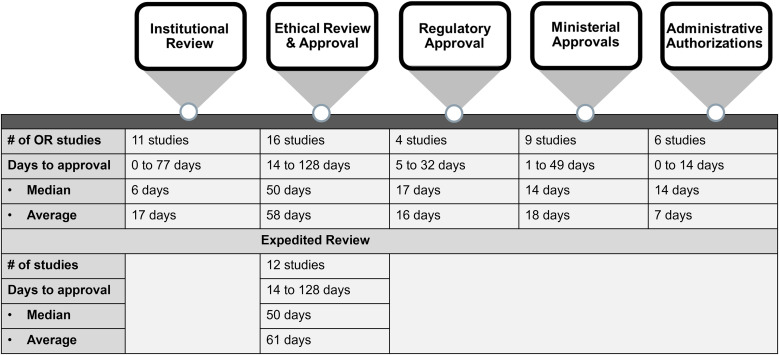
Timelines for study approvals. OR = operational research.

The key barriers for providing timely and relevant evidence to policymakers were the approval timelines and requests to adapt the different research studies to align with the requirements for clinical trials. The approval processes for the 16 OR studies took between 14 and 135 days (median, 77 days) and comprised up to five different steps, including institutional, ethical, regulatory, and ministerial reviews and approval(s) as well as administrative authorization(s) (Figure 4). The timelines for institutional, regulatory, and MoH reviews and approvals and the timeline for administrative authorizations were each relatively short (median, 6–17 days); however, when one or more of these steps were required, the total process (excluding ethical approval) still took up to 77 days (median, 20 days). It was therefore challenging to provide timely evidence to policymakers, even without the additional time required for ethical approvals.

The ethical review was by far the most time-consuming process, taking between 14 and 128 days (median, 50 days). Expedited review was available for 14/16 OR studies and was used by 12 study teams. Nevertheless, even when expedited review was available and used, this did not reduce the review timelines. Study teams cited the sometimes lengthy review process, frequency of meetings, and lack of familiarity with OR as the main reasons for the significant delays. There was a consensus among study team leads that the review and response timelines could have been shortened if they had been invited to join the ethics committee meeting after their closed session. Speaking directly with members of the ethics committee could provide a valuable opportunity to explain the study, clarify any issues, and immediately address any concerns (Box 6).^11^^,^^12^ Although these clarifications would still need to be provided in writing, it is likely that opportunities for real-time verbal exchanges would have made a second ethics committee meeting unnecessary. This would have significantly shortened approval timelines, considering that the implicated ethics committees met only every 7–60 days (median, 30 days).

Box 6 **Approval in cameroon could be expedited by participating in an ethics committee meeting**

The Elizabeth Glaser Pediatric AIDS Foundation Cameroon team evaluated two different SARS-CoV-2 testing strategies for mass gathering events. The first study was conducted in fan zones during the 33rd African Football Cup of Nations (AFCON), whereas the second study explored SARS-CoV-2 rapid test acceptability and positivity in community gatherings in Cameroon. The ethics committee approval for the AFCON study was received within 15 days, whereas approval for the SAFE protocol took 3 months. The principal investigator of SAFE was invited to present the study and directly responded to queries at the second ethics committee meeting, thereby eliminating the need for a third round of reviews.^11^^,^^12^

Regulatory approvals were not required for 11/16 OR studies because the Ag-RDTs used in the study were commercially available in-country and in all but one study were used per manufacturer’s instructions. For the study that evaluated self-testing, which had not been fully evaluated in Tanzania at the time, all relevant approvals, including the additional approvals needed for self-testing, were received within 54 days.^18^

Overall, the utility of the evidence provided from these studies to policymakers was severely tempered not only by the delays in the various approval processes but also by the changes requested by some ethics committees. We do not question nor would we try to circumvent the essential duty of ethics committees to protect the rights and welfare of research subjects and to ensure that the studies are scientifically sound and of benefit for the study population. Indeed, requests relating to clarifications with regard to confidentiality, more information on the risks and benefits of the testing approach, and suggestions on how the studies could be improved were much appreciated. Nevertheless, we feel that several changes and processes unnecessarily negatively impacted the rapid implementation and utility of much-needed OR during the outbreak, the results of which could have had significant benefit to outbreak control. Specifically, some requests relating to changes to written informed consent (six studies), patient follow-up (four studies), provision of incentives for testing (two studies), additional data collection (one study), and additional objectives (one study) added complexity and costs to various studies that aimed to evaluate a public health approach rather than the diagnostic itself, and thus would generally not be considered clinical trials.

In addition, ethics committee requests for payment of study participants and the need for written informed consent potentially introduced bias into some studies; being paid for testing would logically attract participants, but would limit extrapolation to more real-world settings in which the advantage of remuneration would not exist. Requiring written informed consent for testing with a diagnostic assay that has already been granted full regulatory approval risks participant misunderstanding. Specifically, participants may perceive that the test itself is still being evaluated, with unknown risks and benefits, which may deter participation in the study. In addition, because time required for testing was one of the key factors that deterred individuals from getting tested,^9^^–^^11^^,^^16^ the added time required for the informed consent process would have deterred some people from testing. That said, we fully concur that both written consent and assent should be sought for OR when testing of minors is proposed and that written informed consent is essential when personal data are collected and stored or when repeat testing is proposed.

In summary, we feel that the criteria for clinical trials should not be applied for OR that aims to optimize the testing strategies with approved diagnostics. Evaluating these types of studies as clinical trials adds complexity and time and potentially introduces serious bias, thereby affecting the scientific integrity of the study. International guidelines for ethics committee review of OR, especially if aimed at public health emergencies, are hence urgently needed to streamline and speed the process for the benefit of all.

### 6. Skilled and motivated workforce.

Overall, the study teams involved in this work were highly skilled and committed, with only 1/17 study teams experiencing notable workforce challenges.^6^ Nevertheless, establishing and maintaining a committed, well-versed, and competent workforce willing and able to engage with and operate within communities takes time and resources. This workforce often cannot be easily mobilized in outbreak situations, where demands for skilled labor are high and time for training and capacity for close supervision may be limited.^11^ In these situations, on-site supervision can be complemented with remote supervision via digital tools that, because they reduce travel times, make supervision less time-consuming, cheaper, and more environmentally friendly.^17^

The acceptability of community-based testing relied heavily on trust between the communities and service providers and appeared to rely less on advanced qualifications of individual staff members. For instance, the studies taking place in Mali and Suriname both used community health workers to conduct COVID-19 testing. These community health workers were members of the community who received basic training with regard to malaria and COVID-19 messaging, diagnosis, contact tracing, and basic treatment and care, as well as current referral mechanisms.^14^^,^^17^ The integration of both diseases was well received by the community health workers, improving malaria health outcomes in Mali, where their remit had previously been more restricted.^17^

When staffing challenges were encountered, they existed prior to the pandemic and were exacerbated by COVID-19 fatigue, adverse weather conditions, and outbreaks of violence in the settings where testing was done.^6^ Staff safety was paramount, and where needed, studies were stopped, interrupted,^6^ or adapted^17^ in response to concerns over security or recent events (Box 7).^6^

Box 7 **Safety concerns in low-income communities in Jamaica**

Novamed conducted Ag-RDT testing in three high-risk populations in Jamaica, including underserved and volatile communities. These communities are characterized by a deeply entrenched mistrust in health policies and authorities. Intensive community engagement and close collaboration with respected local entities such as churches were required to build trust. In addition, the team worked closely with the police force and, with agreement of the funder, ceased all activities during an outbreak of violence to ensure the safety of staff and participants.^7^

## CONCLUSION

Well-designed and rapidly implemented OR has the potential to provide essential practical information to policymakers to combat disease outbreaks, but implementation during these often quickly evolving events can be challenging. Outbreaks, especially of novel pathogens, are unpredictable, and transmission dynamics are even more likely to change if the pathogen is prone to frequent mutations, such as with SARS-CoV-2 virus. Research that aims to evaluate strategies for curbing transmission must hence be easily and swiftly adaptable. This requires flexibility from researchers, funders, staff conducting the studies, and ethics and other approval committees. Based on our collective experience performing OR in the often challenging context of the COVID-19 pandemic, we have outlined several areas of importance and gaps to be addressed and made recommendations toward that end (Figure 1 and Box 8). Although numerous factors enabled the generation of evidence for policymakers during the COVID-19 pandemic, several structural aspects will need to be adjusted to provide more timely and relevant evidence to policymakers during future disease outbreaks. We recommend that the requirements described in Box 8 be implemented to the fullest extent possible to improve the impact of OR during epidemics and pandemics.

Box 8 **Key requirements for or to inform local policy in disease outbreaks**

1. Operational research should be aligned with priorities of the community and local and national government authorities.
2. Close communication and collaboration between the key stakeholders, as well as technical partners, is essential to success.
3. Approval processes need to be streamlined and shortened while still ensuring ethical conduct, sound scientific practice, and accountability.
4. Mechanisms should be created for swift reallocation of funds when necessary, while maintaining accountability.
5. Flexibility that allows for the research to be adapted to changing disease and political dynamics must be ensured.
6. Research results should be disseminated in a timely manner to all stakeholders and, where applicable, rapidly incorporated into policy and practice.

## Supplemental Materials

10.4269/ajtmh.23-0893 Supplemental Materials
