# Supplementary material for: Operational Research to Support Rapid Evidence-Based Responses to Outbreaks: Learnings from COVID-19
Source: Am J Trop Med Hyg. 2024 Oct 8;112(4 Suppl):119–26. doi: 10.4269/ajtmh.23-0893 (PMC11965719; doi:10.4269/ajtmh.23-0893)
Supplement: Supplemental Materials [file tpmd230893.SD1.pdf]

SUPPLEMENTARY TABLE 1: STUDY SUMMARIES

Title of Study	Country	Setting	Study Summary	Main Conclusion(s)	Ref.
Optimizing the use of SARS-CoV-2 antigen rapid diagnostic tests for timely detection and response to COVID-19 in schools and markets in Uganda	Uganda	Schools; Markets	The Infectious Diseases Research Collaboration conducted monthly cross-sectional SARS-CoV-2 Ag-RDT surveys in high-risk border communities in Uganda. In total, 10,406 volunteers were tested plus contacts of anyone testing positive.	Routine SARS-CoV-2 Ag-RDT testing in schools and markets is feasible and acceptable. Strategies for encouraging self-isolation will need to be improved.	6
Evaluation of community-based delivery and administration of SARS-CoV-2 Ag-RDTs in Zimbabwe	Zimbabwe	Schools	The Clinton Health Access Initiative (CHAI) implemented the roll-out of SARS-CoV-2 Ag-RDT testing across three schools to support the safe reopening of schools and outbreak surveillance efforts.	This implementation project demonstrated that testing in schools is feasible and acceptable.	NA[1]
Implementation of systematic testing with Ag-RDTs in three different communities in Jamaica – why a different approach is needed	Jamaica	Schools; Healthcare workers; High-risk communities	Novamed conducted weekly Ag-RDT testing i) within low-income high prevalence communities (287 study participants; ≥18 years), ii) of healthcare workers (262 study participants), and iii) within schools (88 study participants; 14 to 17 years).	Weekly testing within high-risk communities is challenging due to the lack of trust in service providers, relatively low health literacy, and outbreaks of violence. These factors need to be addressed now to prepare for future outbreaks.	7
Use of antigen-rapid diagnostic test for detection of COVID-19 cases in university settings in Cameroon	Cameroon	Universities	Centre Pasteur Cameroon tested 7006 volunteers with Ag-RDTs in six state universities in Cameroon, as well as contacts of any positive cases. The team also assessed knowledge, attitude, and practice of this population with regards to COVID-19 infection. A high level (28%) of vaccine hesitancy was reported.	Testing with Ag-RDT in higher education institutions is feasible and acceptable. Additionally, simple restrictions on entry into campus (such as masks) and enforcement of social distancing on campus should be strictly imposed in outbreak situations.	8

Evaluating the acceptability and feasibility of community-based delivery and administration of SARS-CoV-2 AG-RDTs for timely and effective public health response[2]	Malawi; Zambia	Markets	The Clinton Health Access Initiative tested 2348 and 1723 volunteers in marketplaces in Malawi and Zambia respectively to gain a better understanding of the challenges of conducting SARS-CoV-2 Ag-RDT testing in a community setting.	Community testing is a feasible and acceptable intervention to increase testing access in Malawi and Zambia, especially when coupled with community sensitization, and mobilization.	9
Feasibility and Acceptability of SARS-CoV-2 Antigen Rapid Diagnostic Testing in High-Risk Markets and Trade Hubs in Kampala, Uganda	Uganda	Markets; Trade hubs	The Central Public Health Laboratory/Ministry of Health (MoH) Uganda conducted 4 rounds of monthly cross-sectional SARS-CoV-2 Ag-RDT surveys in markets and trade hubs four divisions of Kampala. A total of 13,086 volunteers were tested.	Routine SARS-CoV-2 Ag-RDT testing in markets and trade hubs is feasible and acceptable, with markets being most suitable as testing sites. The time and effort required for testing need to be minimized, and strategies for encouraging self-isolation will need to be improved.	10
Assessing the benefit of two detection approaches in screening COVID-19 using SARS-CoV-2 antigen rapid diagnostic tests among inter-city public bus travelers	Cameroon	Public transport	MA Sante evaluated three different Ag-RDT testing strategies by offering i) systematic COVID-19 RDT testing to all departing intercity passengers, ii) testing to suspected cases only, or iii) no testing. 9594 departing passengers agreed to participate in the study, and 1731 tests were performed. All travelers were called 7-10 days after their trip to identify potential cases.	Offering testing with Ag-RDTs in travel agencies is feasible and acceptable. Approx. 1/3 of passengers consented, and testing did not delay their travel. The approach can detect COVID-19 cases, but novel strategies are needed which increase the proportion of positive cases postponing their travel.	11
Implementing SARS-CoV-2 Ag-RDT at fan zones during large-scale sportive event in Africa: Challenges, opportunities and lessons learned during the 33rd African	Cameroon	Mass gathering events	The Elizabeth Glaser Pediatric AIDS Foundation (EGPAF) supported the Cameroonian MoH with SARS-CoV-2 Ag-RDT testing in fan zones at the 33rd Africa Cup of Nations football tournament. In total, 4,820 fan zone	Testing at mass gathering events needs to be well implemented to capitalize on the benefits of testing while reducing the barriers to testing (such as time required), thereby minimizing the economic impact of such a strategy.	12

Cup of Nations tournament in Cameroon			attendees were tested for SARS-CoV-2.		
Acceptability of community-based, rapid SARS-CoV-2 testing at mass gathering events and community gathering points	Cameroon	Mass gathering events	EGPAF employed community workers to sensitize and refer individuals for COVID-19 Ag-RDT testing to nearby testing points. Overall, 20.5% (2,449/11,945) of sensitized individuals visited testing points and 1,006 individuals were tested for SARS-CoV-2.	Community-based testing was generally perceived as important, but actual testing uptake was still low. For future outbreaks, testing uptake could be improved by reducing misinformation, better mobilization strategies, using alternative testing modalities, and tailoring approaches.	13
A cross-sectional study of the use of Ag-RDTs for community identification of SARS-CoV-2 in Kenya	Kenya	Mass gathering events	EGPAF offered SARS-CoV-2 Ag-RDT testing, vaccination and/or a participation in a survey to 4,062 individuals (≥2 years) attending mass gathering events. Testing acceptance was 78.1%. Community mobilization was the major cost driver (26%) followed by purchase of SARS-CoV-2 Ag-RDT (20.5%). The cost per individual tested was US$15.89.	Targeted mass community testing using SARS-CoV-2 Ag-RDT is a feasible and affordable strategy.	14
Provision of COVID-19 services by Community Health Workers to remote gold mining communities in Suriname	Suriname	Migrants; Remote communities	The Foundation for the Advancement of Scientific Research in Suriname, Social Solutions, and the Malaria Program of the MoH worked with Community Health Workers (CHWs) to integrate COVID-19 diagnostic and treatment services into their existing malaria program. In total 1300 persons were tested for SARS-CoV-2 and 8% of these tested positive.	The project demonstrated the feasibility and benefits of integrating COVID-19 testing into the CHWs malaria services. It helped to lower health access barriers in these difficult-to-reach populations in the remote interior of Suriname.	15, 20
Evaluating the effectiveness and	Thailand	Migrants	Community Health Volunteers, deployed by the Shoklo Malaria	The community-based SARS COV-2 Ag-RDT testing strategy is	NA[1]

feasibility of SARS-CoV-2 Ag-RDT in the Myanmar migrant community in Thailand for controlling coronavirus transmission			Research Unit, tested 263 individuals with COVID-19 symptoms plus 41 contacts of COVID-19 patients for SARS-CoV-2 using Ag-RDTs.	feasible with necessary support (such as food) during periods of self-isolation.	
COVID-19 community testing centers impacted on testing volume and reaching high-risk population in Maputo City and province, Mozambique	Mozambiqu e	Markets; Ports	The National Institute of Health in Mozambique assessed the effectiveness of a walkthrough COVID-19 testing approach for reaching high-risk populations that do not attend health facilities. Overall, 4,453 individuals were tested (59.9% in markets and 40.0% in ports). 70.0% of individuals were fully vaccinated.	The walkthrough testing approach is effective in reaching populations who do not traditionally attend health facilities and could be used for screening for comorbidities, surveillance activities and during outbreak situations.	16
Examining psycho-social determinants influencing COVID-19 testing uptake in urban and rural South African communities.	South Africa	Communities	IRD and the University of Cape Town tested 3074 adults for SARS-CoV-2 with Ag-RDT in community settings. Additionally, 704 individuals participated in a perceptions survey which revealed that men, older adults, and urban populations were less likely to undergo COVID-19 testing.	Testing rates can be improved through targeted health campaigns, but larger-scale implementation research is required to refine best-practices.	17
COVACOM: optimizing the control of COVID-19 by introducing SARS-CoV-2 Ag-RDTs at the community level in Mali	Mali	Communities	Solthis compared a community-based, integrated SARS-CoV-2 and malaria testing strategy to the national strategy in four health areas of the Fana district. In the intervention arm, CHWs tested 386 patients with symptoms of COVID-19 and/or fever for SARS-CoV-2 and/or malaria using Ag-RDTs.	Integration of community-based testing for SARS-CoV-2 and malaria was shown to be feasible and effective. Ag-RDTs are essential tools for CHWs to tackle pandemic and endemic diseases.	18
Experience gain on deployment of COVID-19	Tanzania	Transport hubs;	Ifakara Health institute offered assisted or self-testing to 531	Assisted and self-testing within the community was well accepted	19

Ag-RDT self-testing in selected communities in Tanzania		Mining communities	symptomatic or SARS-CoV-2 exposed individuals. 84% accepted to be tested, and approximately 1/3 opted for self-testing.	and the approach could increase the coverage of testing within these communities.	

[1]NA=Not applicable; unpublished

[2]Counted as two studies for the purpose of this manuscript

SUPPLEMENTARY TABLE 2: STUDY TEAM MEMBERS

**Central Public Health Laboratories (CPHL), Uganda**	Grace Esther Kushemererwa, Hellen Nansumba, Isaac Ssewanyana*, Julius Simon Otim, Martha Ampumuza, Sam Acellam Acaye, Sarah Zalwango, Susan Nabadda*, Victor Bigira
**Centre Pasteur Cameroon (CPC)**	Abdou Fatawou Modyinyi, , Chavely Gwladys Monamele*, Henri Moumbeket, Landry Messanga , , Richard Njouom*, Ripa Mohamadou, Sylvanus Akoaghe<br><br>*University of Buea*: Bimela Chrysantus Njobinkir, Fondzenyuy Ahmadou Yaya, Jane Francis Akoachere, Judith Christine Eyong Etaka<br><br>*University of Douala*: Ange Demanou Djounessi, Carole Else Eboumbou Moukoko, Florence Avina Albertine, Marie Chantale Bissa, Marie Madeleine Moukouri, Serge Kingue Etame<br><br>*University of Dschang:* Ambroise Tadonguime, Clifford Ajim Nsani Fogue Pythagore, Gustave Simo, Julienne Zoyem<br><br>*University of Maroua:* Armand Abdou Bouba, Augustin Goudoum, Elie Haman, Serge Rapmo<br><br>*University of Ngaoundere*: Caroline Mangwi, Cathy Gwodog Lobe, Dalvanie Sorel Bokob Kom, Nicolas Njintang Yanou, Samuel Mbozo'o Mvondo, Yaphet Fotue<br><br>*University of Yaounde:* Yacouba Foupouapouognigni
**Clinton Health Access Initiative (CHAI), Malawi**	Andrews Gunda*, Chancy Chavula, Fiona Gambanga, Francis Chitanda, Jonathan Mtaula, Joseph Makondesa, Shaukat Khan, Tamara Mwenifumbo, Trevor Peter*<br><br>*Ministry of Health*: Joseph Bitilinyu-Bangoh*
**Clinton Health Access Initiative (CHAI), Zambia**	Fiona Gambanga, Lindiwe Nchimunya, Namwaka Mulenga, Yucheng Tsai, Shaukat Khan, Trevor Peter*<br><br>*Ministry of Health:* Aaron Shibemba*, Powell Choonga
**Clinton Health Access Initiative (CHAI), Zimbabwe**	Bongani Dube, Brian Chitungo, Ciru Wanjiru Ndichu, Fiona Gambanga, Nevielle Makaka, Shaukat Khan, Tatenda Maparo, Trevor Peter*<br><br>*Ministry of Health and Child Care:* Agnes Juru, Lucia Sisya, Raiva Simbi*, Tanaka Sakubani

**Elizabeth Glaser Pediatric AIDS Foundation (EGPAF), Cameroon**<br><br>AFCON Study Team	Appolinaire Tiam, Boris Kevin Tchounga*, Boris Youngui Tchakounte, Cassandra Kelly-Cirino, Epée Emilienne, Eugène Sobngwi, Joseph Fokam, Loic Feuzeu, Michelle Gill*, Patrice Tchendjou, Rhoderick Machekano, Rogacien Kana, Tatiana Djikeussi, Yap Boum
**Elizabeth Glaser Pediatric AIDS Foundation (EGPAF), Cameroon**<br><br>SAFE Study Team	Albert Mambo, Appolinaire Tiam, Boris Kevin Tchounga, Boris Youngui Tchakounte, Charlotte Moussi, Cassandra Kelly-Cirino, Epée Emilienne, Heather Hoffman, Joseph Fokam, Lolc Feuzeu, Michelle Gill*, Patrice Tchendjou, Rogacien Kana, Tatiana Djikeussi*
**Elizabeth Glaser Pediatric AIDS Foundation (EGPAF), Kenya**	Cassandra Kelly-Cirino, Edyth Atieno Osire, Elgiva Marianne Wanyama, Everlyne Adoyo, Julie Anyango Opar, Lilack Achieng Odhiambo, Lise Denoeud, Mario Songane, Nelson Omondi, Rose Masaba*, Rosemary Maeri, Sophie Otieno, Stephen Siamba<br><br>*George Washington University:* Heather Janel Hoffman<br><br>*Kiambu County Ministry of Health:* Carolyne Magoma Mwancha, Teresia Njoki Kimani
**FIND, Switzerland**	Anne Hoppe. Daniel Bausch, Elena Ivanova Reipold, Pallavi Dani, Stefano Ongarello
**Foundation for the Advancement of Scientific Research in Suriname (SWOS), Suriname**	Stephen Vreden<br><br>*Malaria Program, Ministry of Health Suriname:* Hedley Cairo, Hélène Hiwat<br><br>*Social Solutions Suriname:* Marieke Heemskerk
**Infectious Diseases Research Collaboration (IDRC), Uganda**	Charles Toopaco, Emmanuel Arinaitwe, Harriet Namulezi, Isaac Sewanyana, Jane Frances Namuganga, Jerry Mulondo, Joaniter I Nankabirwa*, Moses R Kamya*, Patience Nayebare, Paul Okoth Odeyo, Paul Olowo, Sally Geoffrey Otema, Sonita Nalukenge, Susan Nayiga, Valentine Owor, Victoria Magero, Winnie Nuwagaba, Yiga Twaha, IDRC administration team
**Ifakara Health Institute (IHI), Tanzania**	Ali Mohammed, Grace Mwangoka, Mwifadhi Mrisho, Muhidin Mahende, Michael Mihayo, Samson Kihwele, Seif Bakari, Salim Abdulla*, Tunu. Ndanzi, Winifrida Recco<br><br>*National Institute for Medical Research*: Paul Kazyoba
**Instituto Nacional de Saúde (INS), Mozambique**	Denise Chitsondzo Langa, Jorfélia Chilaúde, Júlia Sambo, Júlio Rafael, Nádia Sitoe*, Nédio Mabunda, Neuza Nguenha, Phath Guambe

Clinton Health Access Initiative: Chishamiso Mudenyanga, Osvaldo Loquiha

IRD, South Africa

Aamir Khan*, Amyn Malik, Aneeta Pasha, Anwill Willemse, Ayanda Mkhize, Bulelani Madonsela, Desiree Cupido, Emily Abrams, Fredlin Eyssen, Goodman Sibeko*, Khethokuhle Caluza, Loyiso Mahandu, Lunga Mayekile, Muhammad Schuitema, National Fynn, Nokhanyo Xaba, Noluthando Mantengela, Nombuso Pretty Langa, Nonkululeko Ngcamu, Onaiza Qureshi, Qiniso Khumalo, Rochelle Heradine, Roseline Du Brein, Sikhumbuzo Sithole, Thembela Luthuli, Thembisile Mthembu, Theresa Maart, Wendy Zuma, Wenzile Dlamini

Meilleur Acces aux Soins de Santé (MA Sante), Cameroon

Claudine Sen Henriette Ngomtcho, Collins Buh Nkum, Donald Kaptue Bopda, Felicité Naah Tabala, Frank Forex Kiadjieu Dieumo, Etienne Guenou, Imelda Sonia Nzinnou Mbiaketcha, Jerome Ateudjieu*, Ketina Hirma Tchio-Nighie, Larissa Nsuh, Loic Kongne Choupo, Nelly Celestine Kollo Magang, Oswald Babilla Samjeh, Roddy Yvain Saha

Novamed

Alison Nicholson, Angela Miller, Chavanea Cox, Chika Ozongwu, Danica Thomas, David Walcott*, Jennifer Solomon, John Lindo, Julia Myers, Kristen Collins, Lorraine Ferguson, Monica Smikle, Safiyah Cambridge, Samantha Johnson, Shaquielle Dias, Shenae Douglas, Tawana Grant, Thaon Simms, Tresana Pearson, Vanessa Bailey Higgins, Wynthrop Taylor

Shoklo Malaria Research Unit, Thailand

Arunrot Keereevijit, Bulakorn Tinoi, Dah Dah, El Lwe Htoo, Francois Nosten, Hla Than, Htet Khaing Lu, Kanjana Winyoorat, Laaongsri Niwetphongprai, Ladda Kajeechiwa, Laypaw Archusuksan, Mar Lar Say, Mavuto Mukaka, Mushell Darakamon, Nyein Min Oo, Pan Ei Phyu, Paw K Moo Eh, Pho Thit, Po Po, Pornpimon Wilaisrisak, Primprapaporn Thongdee, San Soe, Saw Blec, Suchat, Thaw Htwe Min*, Wah Wah Poe, Wanitda Wanitda Watthanaworawit

Solthis, Mali

Aboubacar Maiga, Adiara Coulibaly, Cheick Hamala Fofana, Fatou Diawara*, Gabrièle Laborde Balen, Guillaume Breton*; Issouf Maiga, Kadi Sidiki Coulibaly, Luis Sagaon-Teyssier, Namballa Keita, Odé Kanku Kabemba, Sétio Dembele

Mali Community Health Centres (CSCOMs): Moussa Doumbia, Moussa Toure, Oumar Diarra, Sidiki Diabate

Community health workers: Abiba Diarra, Adame Erkan, Alima Traore, Alou Diarra, Aminata Berthe, Astan Coulibaly, Awa Diarra, Bintou Diarra, Boubacar

Camara, Chaka Traore, Diah Diarra, Djénebou Coulibaly, Dramane Samake, Fanta Konate, Founéba Traore, Haoussa Toure, Kadia Diarra, Kaida Fane, Modibo Kinta, Nantenè Kante, Néné Traore, N'golo Coulibaly, Salimata Diarra, Sira Traore, Yah Coulibaly

Community relays: Abdou Coulibaly, Adama Sangare, Adama Soumare, Amadou Diarra, Amadou Tamboura, Baba Fane, Bah Sissoko Bakari Coulibaly, Bakoro Coulibaly, Bakoro Keita, Bakary Sylla, Barakolo Coulibaly, Barakourou Traore, Birama Konate, Daouda Diarra, Daouda Konate, Diango Diarra, Drissa Traore, Fotigui Coulibaly, Issa Traore, Lamine Diarra, Lamine Samake, Lassine Diarra, Lassine Diarra, Lassine Traore, Kountou dit Bakary Traore, Madou Dembele, Madou Soumare, Mamadou Fomba, Moussa Traore, Oumar Diarra, Sadio Magassouba, Sangare,Madou, Santigui Coulibaly, Siaka Diakite, Soungalo Toure, Tahirou Traore, Youssouf Dembele, Zoumana Samake

*Principal Investigator
